# P058 Unmasking paediatric Takayasu arteritis: acute heart failure and isolated left ventricular dysfunction as a critical red flag

**DOI:** 10.1093/rap/rkag113.088

**Published:** 2026-11-03

**Authors:** Irum Awan, Gregory Skinner, Agnela Hall, Chitra Sundaramoorthy, Arani Sridhar, Khulood Khawaja

**Affiliations:** Department of Paediatric Rheumatology, Leicester Children’ Hospital, UHL NHS Trust, Leicester, United Kingdom; Department of Paediatric Cardiology, Leicester Children’ Hospital, UHL NHS Trust, Leicester, United Kingdom; Department of Paediatric Nephrology, Leicester Children’ Hospital, UHL NHS Trust, Leicester, United Kingdom; Department of Paediatric Rheumatology, Leicester Children’ Hospital, UHL NHS Trust, Leicester, United Kingdom; Department of Paediatric Rheumatology, Leicester Children’ Hospital, UHL NHS Trust, Leicester, United Kingdom; Department of Paediatric Rheumatology, Leicester Children’ Hospital, UHL NHS Trust, Leicester, United Kingdom

## Abstract

**Introduction:**

Pediatric large-vessel vasculitis is frequently underrecognised due to its insidious onset and heterogeneous presentation (1).Takayasu arteritis (TA) emerges as the predominant form (2,3), characterised by widespread, progressive inflammation of the aorta and its major branches (4,5). While cardiac complications typically involve coronary arteries or aortic root disease, isolated left ventricular (LV) dysfunction remains a severe, under-documented manifestation (6). This case report presents an adolescent with life-threatening acute heart failure secondary to isolated LV dysfunction, highlighting the critical role of timely immunosuppression and multidisciplinary care to prevent morbidity and mortality (7).

**Case description:**

A 16.5-year-old female with Attention-Deficit Hyperactivity Disorder (ADHD) presented with a 4-month history of mild breathlessness progressed to orthopneoa, hemoptysis, and weight loss. She was admitted for acute heart failure revealed pulmonary oedema, hypertension, and elevated pro-BNP(3878). Echocardiography confirmed severe left ventricular LV dysfunction (fractional shortening ∼20%). Although her anaemia was initially linked to menorrhagia, a paradoxical hypertensive response to ACE inhibition prompted rheumatology referral. Initial autoimmune screens were negative; however, notably limb blood pressure discrepancies and severe LV dysfunction mandated urgent magnetic resonance angiography (MRA). This confirmed TA with critical aortic stenosis and extensive renal and mesenteric involvement. Her carotid doppler and brain imaging excluded intracranial vasculitis or thrombotic complication.

Our patient exhibited high disease activity (PVAS 18/63) and was treated with pulse methylprednisolone twice and weekly subcutaneous methotrexate. Persisting symptoms led the rheumatology team to initiate biological therapy. Following a rituximab-induced hypersensitivity reaction, the team transitioned her to tocilizumab, which facilitated clinical stabilisation and LV systolic function improvement to 45%. A “Hospital Passport” was utilised to accommodate ADHD-related needs. PET imaging confirms quiescent disease.

She continued the MDT care closely, including cardiology and renal team. She received three months of tocilizumab for two weekly IV doses. She was discharged home with oral prednisolone with gastro-protection, carvedilol, aspirin, amlodipine and diuretics. She demonstrated clinical improvement (PVAS 2/63), however chronic renovascular changes persist. Interventional radiology performed successful renal artery angioplasty in an age-appropriate super-specialised centre, achieving normalized blood pressure. Her most recent PET scan shows no active vasculitis. Currently she is on weekly S/C tocilizumab with transition care in progress.

**Discussion:**

This case highlights isolated left ventricular cardiac dysfunction as a rare but critical presentation of paediatric TA. Clinical assessment must be rigorous; practitioners should maintain a low threshold for suspecting large-vessel involvement in patients with nonspecific constitutional symptoms and unexplained cardiac dysfunction. Bedside skills, particularly the routine assessment of differential limb blood pressures, remain essential for early diagnosis.

Advanced imaging, specifically PET-CT or MRA, is paramount for identifying granulomatous inflammation often missed by standard laboratory screening. Treatment pathways require nuanced decision-making. When standard heart failure pharmacotherapy and antihypertensives are inadequate, structural interventions like angioplasty are necessary to manage blood pressure. Biologic therapy with IL-6 inhibitors tocilizumab is highly effective for refractory cases. Clinician oversight is required to manage hypersensitivity and drug-related side effects.

Finally, managing paediatric patients with comorbidities like ADHD requires proactive adaptation. Extended clinic hours, clear communication, and the support of specialist nurses were critical in ensuring treatment compliance and out patient well-being

**Key learning points:**

• Differential Diagnosis: Consider Takayasu arteritis in unexplained paediatric heart failure with isolated LV dysfunction, particularly when systemic hypertension is present.

• Early Screening: Evaluate constitutional symptoms (weight loss, anaemia) earlier to avoid delays in management, as early-stage heart failure or coronary involvement is associated with poorer disease progression.

• Physical Assessment: Perform routine differential blood pressure readings in all patients with unexplained cardiac dysfunction.

• Imaging: Utilize MRA and PET-CT for prompt detection and active disease monitoring.

• MDT Approach: A multidisciplinary collaborative management is essential for optimizing long-term cardiac, renal, and psychological outcomes.

• Therapeutics: IL-6 receptor antagonists like Tocilizumab are effective for inducing and maintaining remission in paediatric cohorts.

• Neurodiverse Adaptation: Clinical management must be adapted for neurodivergent patients to ensure adherence and minimize distress.

**References:**

1. Schnabel A, Hedrich CM. Childhood Vasculitis. Frontiers in Pediatrics. 2019;6: 421–421.

2. Evers K, Eleftheriou D, Brogan P, Marlais M, Tullus K. #3795 Childhood Takayasu arteritis: a multicentre retrospective cohort study. Nephrology Dialysis Transplantation. 2025;40.

3. Mathew AJ, Goel R, Kumar S, Danda D. Childhood‐onset Takayasu arteritis: an update. International Journal of Rheumatic Diseases. 2015;19(2): 116–126.

4. Russo R, Katsicas MM. Takayasu Arteritis. Frontiers in Pediatrics. 2018;6: 265–265.

5. Brunner J, Feldman BM, Tyrrell PN, Kuemmerle‐Deschner J, Zimmerhackl LB, Gaßner I, et al. Takayasu arteritis in children and adolescents. Lara D. Veeken. 2010;49(10): 1806–1814.

6. Hadi A, Rahman MA, Utamayasa IKA, Hidayat T, Wicaksono H, Nastiti PH. Takayasu arteritis in pediatric: a rare case. Romanian Journal of Pediatrics. 2025;74(4): 273–276

7. Eleftheriou D, Varnier G, Doležalová P, McMahon AM, Al-Obaidi M, Brogan P. Takayasu arteritis in childhood: retrospective experience from a tertiary referral centre in the United Kingdom. 2015; Arthritis Res Ther 17, 36 (2015)

